# Molecular profiling of an osseous metastasis in glioblastoma during checkpoint inhibition: potential mechanisms of immune escape

**DOI:** 10.1186/s40478-020-00906-9

**Published:** 2020-03-09

**Authors:** Malte Mohme, Cecile L. Maire, Simon Schliffke, Simon A. Joosse, Malik Alawi, Jakob Matschke, Ulrich Schüller, Judith Dierlamm, Tobias Martens, Klaus Pantel, Sabine Riethdorf, Katrin Lamszus, Manfred Westphal

**Affiliations:** 1 Department of Neurosurgery, University Medical Center Hamburg-Eppendorf, Martinistr. 52, 20246 Hamburg, Germany; 2 Department of Oncology and Hematology, Bone Marrow Transplantation with Section Pneumology, Hubertus Wald University Cancer Center Hamburg, University Medical Centre Hamburg-Eppendorf, Hamburg, Germany; 3 Institute of Tumor Biology, University Medical Center Hamburg-Eppendorf, Hamburg, Germany; 4 Bioinformatics Core, University Medical Center Hamburg-Eppendorf, Hamburg, Germany; 5 Institute of Neuropathology, University Medical Center Hamburg-Eppendorf, Hamburg, Germany; 6 Department of Pediatric Hematology and Oncology, University Medical Center Hamburg-Eppendorf, Hamburg, Germany; 7 Research Institute Children’s Cancer Center Hamburg, Hamburg, Germany

**Keywords:** Glioblastoma, Metastasis, T-cells, Tumour-infiltrating lymphocytes, Immune escape, Whole genome sequencing, Circulating tumour cells

## Abstract

Peripheral metastases of glioblastoma (GBM) are very rare despite the ability of GBM cells to pass through the blood-brain barrier and be disseminated through the peripheral blood. Here, we describe a detailed genetic and immunological characterization of a GBM metastasis in the skeleton, which occurred during anti-PD-1 immune checkpoint therapy. We performed whole genome sequencing (WGS) and 850 K methylation profiling of the primary and recurrent intracranial GBM as well as one of the bone metastases. Copy number alterations (CNA) and mutational profiles were compared to known genomic alterations in the TCGA data base. In addition, immunophenotyping of the peripheral blood was performed. The patient who was primarily diagnosed with *IDH*-wildtype GBM. After the resection of the first recurrence, progressive intracranial re-growth was again detected, and chemotherapy was replaced by PD-1 checkpoint inhibition, which led to a complete intracranial remission. Two months later MR-imaging revealed multiple osseous lesions. Biopsy confirmed the GBM origin of the skeleton metastases. Immunophenotyping reflected the effective activation of a peripheral T-cell response, with, however, increase of regulatory T cells during disease progression. WGS sequencing demonstrated distinct genomic alterations of the GBM metastasis, with gains along chromosomes 3 and 9 and losses along chromosome 4, 10, and 11. Mutational analysis showed mutations in potentially immunologically relevant regions. Additionally, we correlated tumour-infiltrating lymphocyte and microglia presence to the occurrence of circulating tumour cells (CTCs) in a larger cohort and found a decreased infiltration of cytotoxic T cells in patients positive for CTCs. This study exemplifies that the tumour microenvironment may dictate the response to immune checkpoint therapy. In addition, our study highlights the fact that despite an effective control of intracranial GBM, certain tumour clones have the ability to evade the tumour-specific T-cell response and cause progression even outside of the CNS.

## Introduction

Metastatic dissemination of glioblastoma (GBM) is rare. Less than 0.5% of patients develop extracranial GBM tumour manifestation [12, 20, 31, 32]. Multiple hypotheses have been postulated why, this otherwise so aggressively growing tumour, only rarely forms metastases outside the brain. The commonly discussed pathophysiological ideas are the “*seed vs soil*” hypothesis [16, 29], which describes the inability of cells to adapt and home to the tissue microenvironment outside the brain, and the “*peripheral immunosurveillance*” hypothesis, which holds that the activated peripheral immune system is able to eliminate GBM tumour cells that left the immune protected brain microenvironment.

The discovery of circulating tumour cells (CTCs) in GBM has renewed the interest in this discussion. In one of the first studies of its kind, Müller et al. detected CTCs in up to 20% of patients [31]. Cells in the peripheral blood were identified by their expression of the astrocytic marker GFAP. Further studies incorporating additional CTC isolation techniques consecutively found circulating GBM cells in up to 39% of patients, with a preferential enrichment of CTCs in GBM displaying a mesenchymal gene expression profile [24, 39]. It remains unclear, however, why the CTCs do not form extracranial tumours at the expected frequency. The occurrence of extracranial GBM metastases in patients receiving organ donations from GBM patients points to a decisive role of the immune system in containing extracranial growth [19].

GBM metastases have been described to occur at various sites, including bone, lymph nodes, lung and liver, but also to the skin, thus in part challenging the “seed vs soil” hypothesis [14, 15, 17, 34, 41]. In the context of ongoing trials to study the efficacy of immunotherapy for GBM, recent studies focused on the immunosuppressive capacities of GBM in the brain microenvironment as well as its immunomodulation of the peripheral immune surveillance [5, 9, 30]. These studies revealed a strong interaction of the intracranial tumours with the peripheral immune system and demonstrated that immune escape of GBM is not limited to the local tumour environment, but also impacts the peripheral immune system. These observations emphasize the importance to study immune escape mechanisms in rare cases of extracranial GBM metastasis.

In the here presented case, we illustrate the immunological escape of a peripheral GBM metastasis, which formed during intracerebral tumour control with checkpoint inhibition. The molecular and immunological profiling presented in this study provides new insights into potential mechanisms of immune escape and metastatic tumour evolution.

## Case presentation

**Fig. 1:**
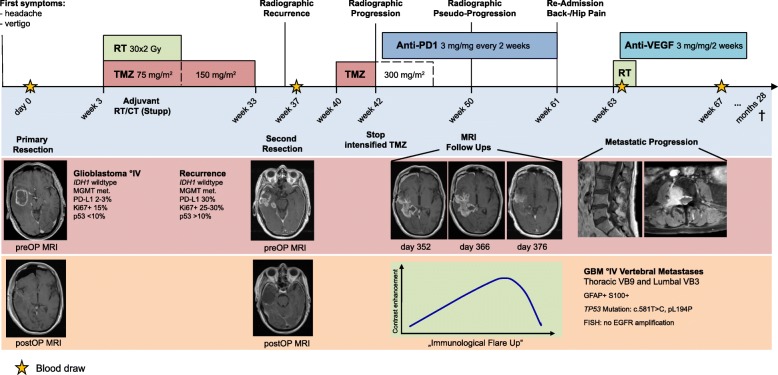
Clinical time-course. The first row illustrates the different therapeutic interventions. The yellow star highlights the collection of peripheral blood for immune cell analysis. Magnetic resonance images (MRI) of key events during disease progression, as well as histopathological results, are shown below. RT: radiotherapy, TMZ: temozolomide, VB: vertebral body

**Fig. 2:**
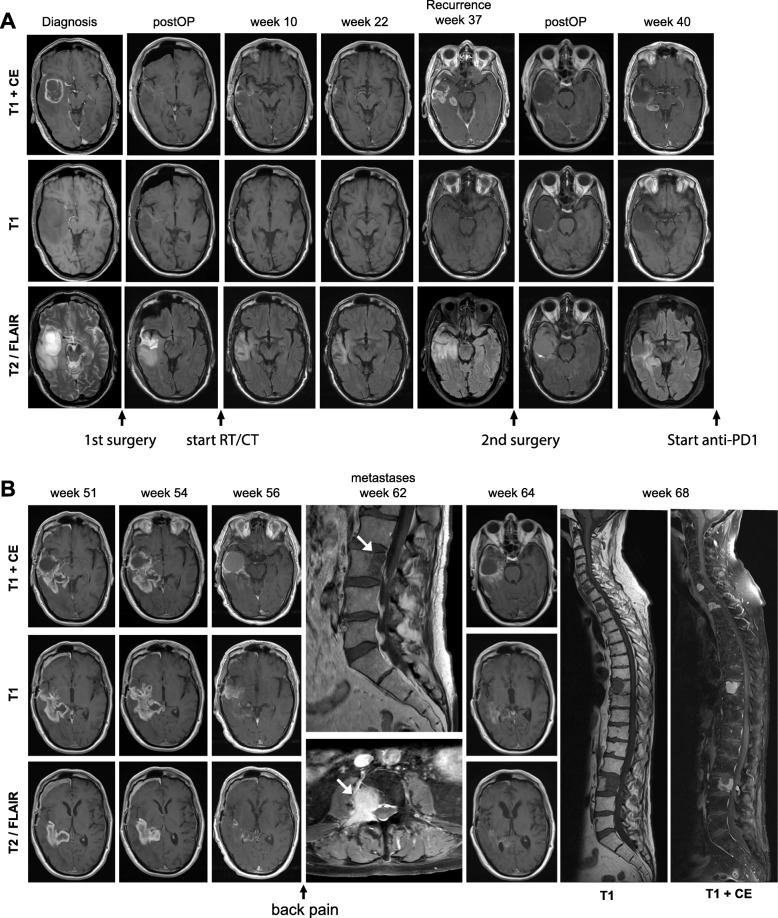
MR imaging and time-course. **a** MRI at various time-points during disease progression until anti-PD1 was administered. **b** Intracranial flare-up and remission after initiation of anti-PD1 treatment, and occurrence of multiple extracranial GBM metastasis while the intracranial tumours remained stable (week 64). White arrows show the intraosseous GBM metastases

**Fig. 3:**
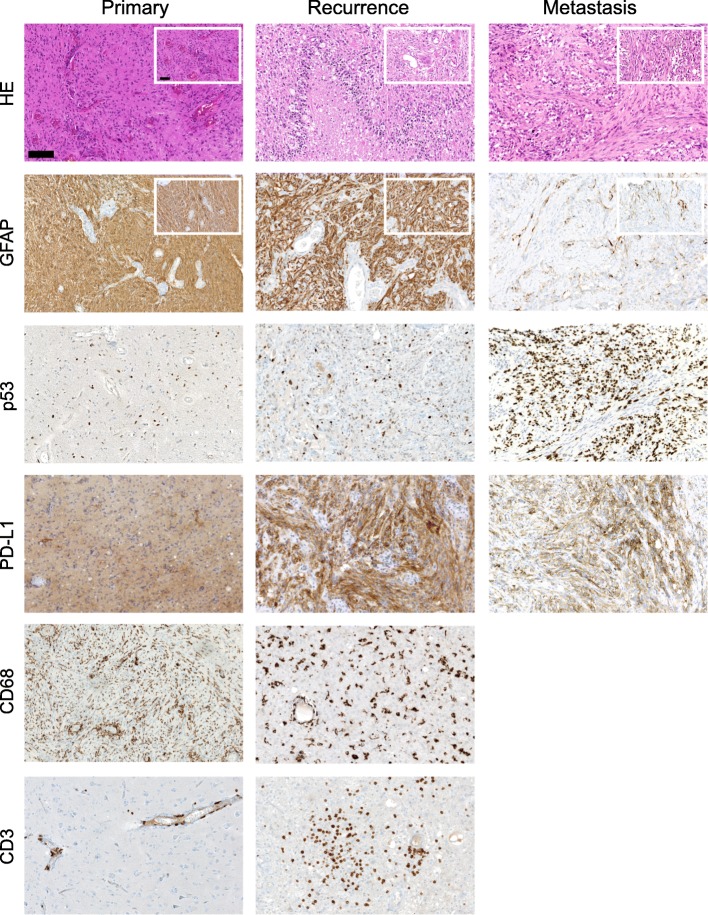
Histological and immunohistochemical analyses. Staining for hematoxylin & eosin (HE), p53, glial fibrillary acidic protein (GFAP), PD-L1, CD68 (microglia) and CD3 (pan T cell) of the initially diagnosed GBM (primary), its first intracranial recurrence (recurrence) and the biopsy specimen of the vertebral body metastasis from L3 (metastasis). Scale bars indicate 100 μm in larger images and 50 μm in insets

A 74-year-old male patient initially presented with dysphasia, vertigo and fatigue. Cranial MR imaging revealed a large contrast-enhancing inhomogeneous mass in the right temporal lobe (Fig. 1). Surgical resection and subsequent histological analysis and methylation array analysis confirmed the presence of an *IDH1*-wildtype GBM WHO °IV with methylated *MGMT* gene promoter and mesenchymal subtype. Standard adjuvant therapy with combined radio-chemotherapy with temozolomide and 30 × 2 Gy was initiated (Fig. 1). In week 37 a recurrence was detected (Fig. 2). Second resection confirmed active tumour recurrence with strong PD-L1 expression (30%) (Fig. 3). The interdisciplinary tumour board recommended intensified temozolomide therapy. Shortly after initiation of the chemotherapy, large radiographic progression was detected (Fig. 2). Given the fast progression, high PD-L1 expression in the recurrent tumour (Fig. 3), and the at the time ongoing phase-III nivolumab trials, [33] anti-PD1 checkpoint inhibition with nivolumab was initiated. Within 4 weeks the contrast-enhancing lesion increased (Fig. 2). Due to a stable clinical appearance of the ambulatory patient, nivolumab treatment was continued. Short term MR imaging then showed an almost complete remission of the intraparenchymal contrast enhancing lesion, which was suspected as immunological flare up and response to checkpoint inhibition (Fig. 2b). Unfortunately, 6 weeks later the patient came back with severe back pain. Whole-spine imaging demonstrated multiple intraosseous enhancing lesions in vertebral bodies C7, Th2, − 9 and L3. Needle biopsy of L3 and interdisciplinary pathological evaluation, together with the presence of GFAP positive cells and the absence of epithelial (e.g. AE1/3, EMA) and melanocytic (e.g. S100, HMB45, Melan-A) markers confirmed metastatic dissemination of the intracerebrally controlled GBM (Fig. 3). The Ki67-labeling index was positive in 10–15% of cells. Spiral computer tomography (CT) of the thorax and abdomen did not show other masses suspicious for another cancer entity. Nivolumab treatment was stopped and radiotherapy of the spinal tumours combined with anti-angiogenic treatment using bevacizumab was started (Fig. 1). Throughout the radiotherapy the patient further progressed, and the general health condition decreased. In the palliative context, treatment was discontinued, and the patient died 28 months after the initial diagnosis.

**Fig. 4:**
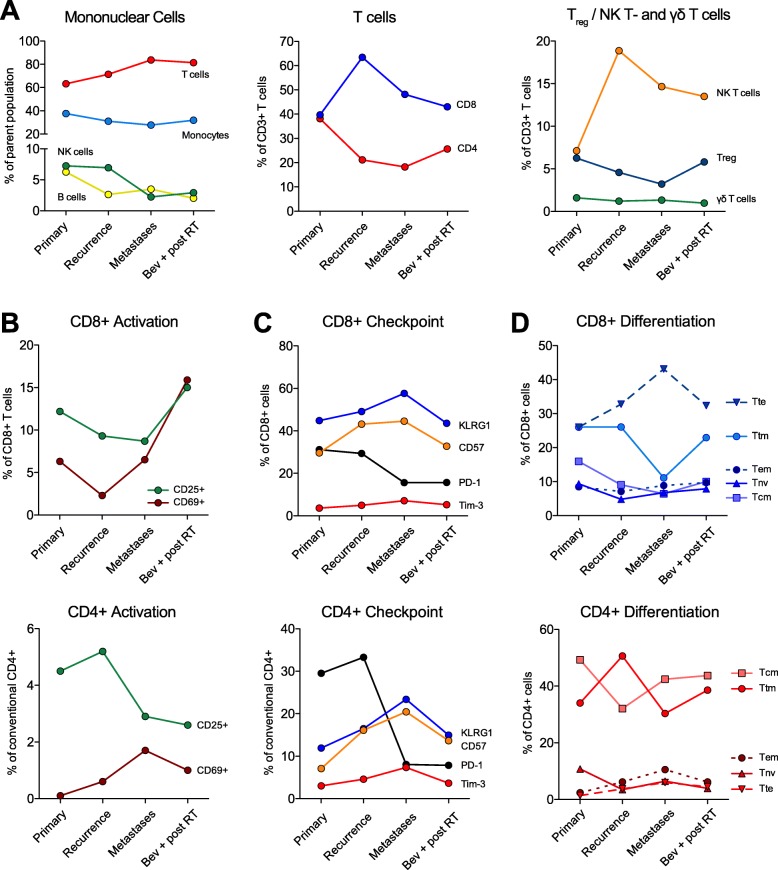
Immune phenotyping of the peripheral blood during disease evolution. **a** Overview of the main immune subpopulations (left panel), CD4/CD8 ratio (middle panel) and specific T cell subsets (right panel). **b** T cell activation of CD8^+^ (upper) and CD4^+^ (lower panel) T cells, as defined by their surface expression of the high affinity IL-2 receptor (CD25) and CD69. **c** Expression of checkpoint molecules Killer cell lectin-like receptor subfamily G member 1 (KLRG1), CD57, programmed death-1 (PD-1) and T-cell immunoglobulin and mucin-domain containing-3 (Tim-3) on CD8^+^ (upper) and CD4^+^ (lower panel) T cells. **d** T cell differentiation defined by CD45RA/CCR7/CD28 marker expression. T_NV_ = naïve (CD45RA^+^, CCR7^+^, CD28^+^), T_CM_ = central memory (CD45RA^−^, CCR7^+^, CD28^+^), T_TM_ = transitional memory (CD45RA^−^, CCR7^−^, CD28^+^), T_EM_ = effector memory (CD45RA^−^, CCR7^−^, CD28^−^), T_TE_ = terminal effector (CD45RA^+^, CCR7^−^, CD28^−^)

Given the unique pattern of intracranial remission during checkpoint inhibition and the simultaneous metastatic peripheral osseous dissemination, further immunological and genomic profiling was performed. Phenotyping of the peripheral blood immune subpopulations at the time of initial tumour resection, tumour recurrence, metastatic presentation and during further adjuvant therapy revealed a steady increase in the T cell population. This increase was dominated by a CD8^+^- and NK T cell peak during the first intracerebral tumour recurrence, while regulatory T cells dropped continuously until occurrence of metastases before again increasing in the final disease stage (Fig. 4a). CD8^+^ T cell activation, as reflected by CD25 surface marker expression, declined until radiotherapy of the vertebral body metastases was performed (Fig. 4b). Whereas the expression, or more likely detectability, of the immune checkpoint marker PD-1 significantly dropped after nivolumab treatment was started, the expression of other immune checkpoint molecules, such as KLRG1, CD57 and Tim-3 increased until peripheral metastasis occurred. After radiotherapy of the spinal metastases and during anti-angiogenic treatment, their expression again decreased (Fig. 4c). The increase of “exhaustion” markers, which are expressed during the process of T-cell activation before metastasis, was accompanied by an increase in the CD8^+^ terminal effector (Tte) T cell compartment (Fig. 4c, d), which describes a memory T cell population with a potentially strong cytolytic function, but limited anti-cancer efficacy due to its inability to self-renew [2]. The CD4^+^ T cell differentiation remained rather stable at all four time-points.

In order to investigate if the patient had peripherally circulating tumour cells (CTCs), we screened the blood of the patients at multiple time points using GFAP immunostainings to identify CTCs, as previously published [31]. In addition, given the p53 mutation in the peripheral metastasis, we also performed p53 immunostainings. No CTCs could be detected at any given time-point in this patient, not even in the metastatic state.

**Fig. 5:**
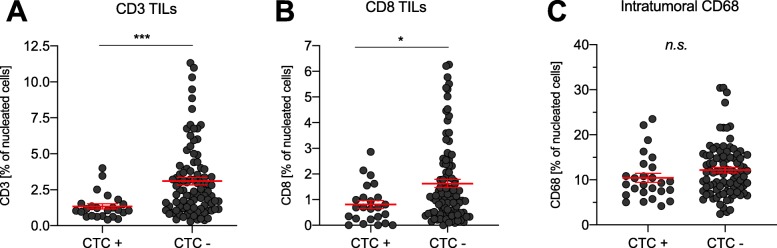
Circulating tumour cell (CTC) count correlation to immunohistochemically quantified tumour-infiltrating immune cells. Presence of tumour-infiltrating lymphocytes (TIL), as automatically quantified by % of nucleated cells was performed for CD3^+^ (**a**) and CD8^+^ T cells (**b**), as well as for CD68^+^ microglia and correlated to the presence of GFAP^+^ peripheral circulating tumour cells, as defined in a previously published cohort [31].

However, to analyse if the presence of CTCs in general could be correlated with the amount of tumour-infiltrating T cells and intratumoural microglia, we immunohistochemically stained the tumours of the previously published cohort for CD3, CD8 and CD68 (*n* = 116, Fig. 5) [31]. Here, the presence of peripheral CTCs (CTC+) correlated with low intratumoural T cells (CD3^+^, *p* = 0.001, *t*-test) and low cytotoxic T cells (CD8^+^, *p* = 0.014, *t*-test), while no difference for CD68^+^ macrophages/microglia was observed (Fig. 5c). This observation highlights the potential link between the tumour-specific immunocompetence and the occurrence of peripheral CTCs.

**Fig. 6:**
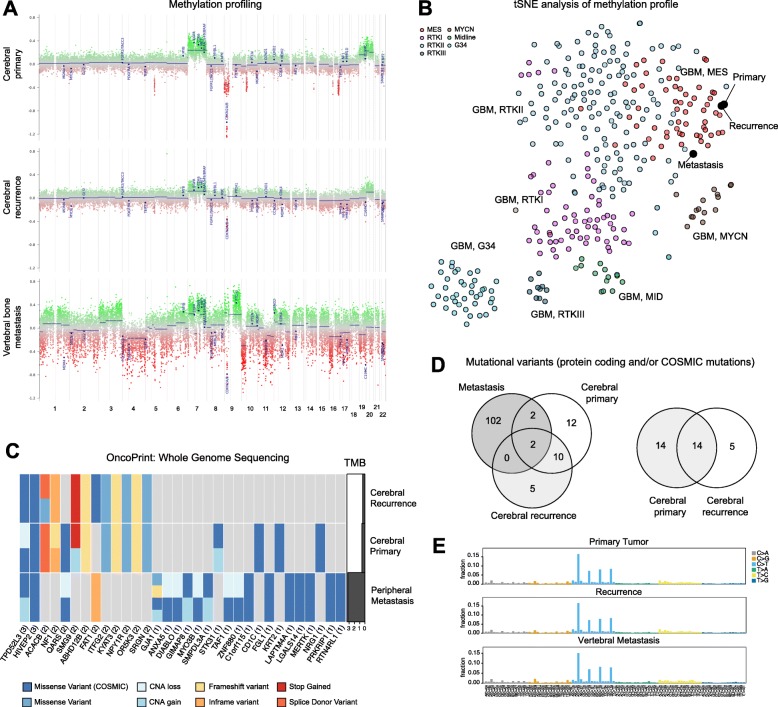
Whole genome sequencing (WGS), copy number alteration- and 850 K methylation profiling of extracranial GBM metastasis. **a** CNA profile obtained from 850 K methylation profiling confirms the WGS profile (Suppl. Fig. 1) and highlights unique molecular profile of the extracranial metastasis. **b** tSNE clustering comparing the three tumours with published methylation profiles by Capper et al. restricted to GBM subgroups, show a mesenchymal transcriptional signature of the cerebral tumours, while the metastasis clusters on the border of the mesenchymal (MES) and RTKII cluster. **c** OncoPrint was generated from WGS data of the initially diagnosed GBM (cerebral primary), its first intracranial recurrence (cerebral recurrence) and the biopsy specimen of the vertebral body metastasis from L3. A gene mutation is shown if it is affected in at least two samples or if it has at least one variant with a COSMIC ID. In addition, mutational burden is depicted. **d** The overlap of single nucleotide variants between the three tumours, which affect protein coding regions with a known COSMIC mutation are depicted as a Venn diagram (left). Due to reduced gDNA quality of the peripheral metastasis only variants in regions covered at least 8x in the depicted samples were considered. **e** Cosmic signature analysis shows single base substitution signature type 1 (C > T) in all three tumours. **d** CNV profile obtained from 850 K methylation profiling confirms the WGS profile (Suppl. Fig. 1) and highlights unique molecular profile of the extracranial metastasis. **e** tSNE clustering comparing the three tumors with published methylation profiles by Capper et al. restricted to GBM subgroups, show a mesenchymal transcriptional signature of the cerebral tumours, while the metastasis clusters on the border of the mesenchymal (MES) and RTKII cluster

Next, we were able to isolate genomic DNA from the metastasis biopsy and perform whole genome sequencing (WGS) and 850 K methylation profiling in all three tumour samples. Global genomic methylome confirmed the diagnosis of MGMT-methylated *IDH*-wildtype GBM °IV in all three specimens (calibrated scores: primary GBM: 99, recurrence GBM: 98, metastasis: 89). Copy number alterations (CNA) extracted from the methylation profiling confirmed the WGS analysis with clear amplification of chromosome 7 and deletion of *CDKN2A* in all 3 specimens which are genomic alterations characteristic of GBM (Fig. 6a). However, while the primary and recurrent GBM were grouped into the mesenchymal subtype, the metastasis could not clearly be assigned to the mesenchymal (score 47) or RTK II (score 39) subtype, which is reflected in the t-SNE analysis (Fig. 6b).

WGS analysis also confirmed the GBM origin of the vertebral metastasis, with the CNA profile showing distinct gains in chromosome 7 and losses in parts of chromosome 5 and 9, which were shared between all three tumours (Suppl. Fig. 1). The OncoPrint representation, comparing mutations between all three tumours, shows 32 genes which have been selected if the gene is affected in at least two samples or if it has at least one variant with a COSMIC ID (Fig. 6c, Supplemental Table 1). As the metastasis shares two exclusive mutations with the primary cerebral GBM, we hypothesize that the metastasis clonally derived closer from the primary GBM, rather than from the recurrent GBM (Fig. 6d). However, the exact timing of metastatic manifestation cannot be determined. Mutations in *TPDF52L3* and *HIVEP2* were shared between all three tumours. *HIVEP2*, also known as *MIBP2* (c-myc intron binding protein 1), was not only described to control the expression of multiple genes, many of which are involved in brain development, but also to inhibit glioma growth [38, 40]. A missense mutation might therefore be a common contributor to tumour development in this case. The following alterations characteristic for GBM were detected when applying a less strict threshold for analysis: *PIK3CA* p.E453Q (COSMIC ID: COSM758), NF1 p.I1679_Y1680del (COSMIC ID: COSM6969872) and *CDKN2A*/B (p15/16) deletion, further confirming the GBM origin of the metastasis. Additionally, a new mutation was found only in the metastasis in *TP53* c.581 T > C p.L194P (COSMIC ID: COSM437527) by sanger sequencing during the initial pathological workup, which could be a possible key driver for tumour formation under anti-PD1 treatment. Among others, the metastasis harboured missense variant mutations in immunologically active genes, such as *ANXA5*, *LGALS14* and *FGL1* (Fig. 6a). These mutations have a potential impact on the immune recognition of tumour cells.

While the primary (0.42 mutations per Mbp) and the recurrent (0.60 mutations per Mbp) cerebral tumours, as expected for GBM, displayed low tumour mutational burden (TMB), the vertebral metastasis exhibited a 6.5-times higher mutational burden (3.26 mutations per Mbp) (Fig. 6a, right panel), which is reflected in the greater amount of mutational variants affecting protein coding regions with known COSMIC mutations (Fig. 6d, left Venn diagram, COSMIC mutations only). Overall 28 mutations are found in the primary tumour, of which 14 were also present in the recurrent cerebral tumour, which presented with five new mutations (Fig. 6d, right Venn diagram, all non-silent mutations). The mutational profile was comparable in all the tumours, as they all show a C to T mutational profile characteristic for a COSMIC signature 1 (Fig. 6e) [13].

## Discussion and conclusion

Our study shows a unique case of extracranial metastases from GBM during immunological remission of the intracerebral tumour with checkpoint inhibition. In order to form extracranial metastases, the tumour had to, either gain new genetic drivers to promote peripheral metastatic seeding, or suppress the peripheral immunosurveillance, or develop new mechanisms to evade from immune recognition during its metastatic spread, or both. Multiple case reports of GBM metastases have focused on genetic aberrations, potentially involved in tumour cell manifestation outside the brain [11, 43, 45]. Among others, alterations, such as *BRCA1* and *ARID1A* mutations or overexpression of IGFBP2 have been described in these cases [44, 45]. Although we cannot exactly define when the metastatic seeding in our case occurred or if it is directly linked to anti-PD1 treatment, the tumour became symptomatic and most likely progressed during intracranial remission under immune checkpoint inhibition. We therefore decided to focus our discussion on potential immunological mechanisms.

Recent findings by Congsathidkieth et al. describe that intracranial GBM is able to suppress the peripheral immune response by sequestration of lymphocytes in the bone marrow via a S1P1-mediated mechanism [9]. While this type of immunomodulation might reflect an extension of the unique immunosuppressive intracranial environment in GBM, we do not have strong evidence for this particular phenomenon in the here presented case, as no significant lymphopenia was observed. We rather saw an increase of exhaustion or functional impairment in the T cell compartment, as demonstrated by the increased expression of other checkpoint molecules such as KLRG1, CD57, and Tim-3. This increase might be a result of compensatory mechanisms during PD-1 checkpoint blockade. Similar observations of a compensatory increase or adaptive resistance to PD-1 blockade by upregulation of additional checkpoints was described in mouse model for lung cancer [22]. The observed increase in terminal effector memory CD8^+^ T cells and decrease in the expression of the high affinity IL-2R (CD25) further supports the hypothesis that peripheral immunosurveillance was functionally compromised during the metastatic spread of GBM cells. Potentially relevant is also a recent study by Jiao et al., that demonstrated that the microenvironment of certain organs determines distinct mechanisms of immune escape, as they show that prostate cancer metastasis in the bone utilize TGFβ to restrain T_H_1 differentiation of T cells in order to escape from immune checkpoint therapy [18].

Although CTCs and sometimes even CTC clusters can be found in a large subset of patients, extracranial metastatic outgrowth are very rare in GBM [23, 24, 31, 39]. Interestingly, although to be expected in cases of peripheral metastatic outgrowth, we did not detect CTCs in this case, or a case of an *IDH1* mutated anaplastic astrocytoma WHO °III [26]. It is unclear whether the peripheral immunosuppression of intracranial GBM is insufficient to support extracranial growth of CTCs, or if the CTCs are unable to adapt to the non-CNS microenvironment or unable to establish a similarly suppressive microenvironment under the requirements of a peripheral “soil”. Our observation of significantly reduced overall CD3^+^ T cell and more specific CD8^+^ cytotoxic T-cell infiltration in patients being positive for CTCs indicates a direct link between the tumour-specific immune response, and potentially also immunocompetence, and the occurrence of tumour cells in the circulation.

Even though not reaching the strict definition of a hypermutated genotype, defined by more than 10 mutations per mega base pair, the increased tumour mutational burden of the extracranial GBM metastasis presented in our case, might explain why multiple metastasis were able to form. Although a generally hypermutated phenotype was suggested to favor recognition by tumour-specific immune cells due to the presence of new tumour-specific antigens [10], tumour mutational burden in glioma does not seem follow the usual correlation of a better overall survival or response to checkpoint inhibition with higher mutational burden [1, 36]. It is unclear why a higher mutational burden in glioma rather results in a worsened overall survival [36]. One hypothesis is, that hypermutated tumours decrease the recognizable antigen load below the threshold of effective tumour-specific activation [1, 27, 49].

Another hypothesis favors the occurrence of immunological escape variants by increased prevalence of somatic mutations in the human leukocyte antigen (HLA) class I region [37]. The HLA class I molecules present tumour antigen peptides and complete loss might result in evasion from adaptive immune recognition [37, 48]. Interestingly, somatic mutations in the HLA class I locus were not observed in primary GBM and, although the tissue quality prohibited WGS coverage of the HLA region in the metastasis, we did also not observe mutations of the HLA locus in this case [37]. However, a study by Wang et al. revealed that 15% of recurrent GBM after radio- and chemotherapy with temozolomide presented with a hypermutated genotype [46]. The increased tumour mutational burden might result in additional mutations which might favor immunological escape. The comparably high mutational burden of the studied extracranial metastasis resulted in various somatic mutations in immunologically relevant sites. As described above, we found missense variant mutations in multiple genes, e.g. *ANXA5, LGALS14 and FGL1,* potentially affecting the tumour-specific immune response of the GBM metastasis. *ANXA5*, which also displayed a CNA loss, encodes the protein Annexin V, which is involved in stabilization of the T cell receptor (TCR) and peptide-MHC interactions during formation of an immunological synapse [25]. Galectin-14 (*LGALS14*) is expressed mainly by the placenta and was shown to be able to induce apoptosis of activated T lymphocytes [4]. The gene *FGL1* encodes for a protein called fibrinogen-like protein 1, which was recently described to be highly expressed in many cancers and to inhibit antigen-specific T cell activation through the interaction with the known checkpoint molecule Lag-3 [47]. Increased levels of FGL1 in serum was associated with increased resistance to anti-PD1 checkpoint inhibition [47]. Although the confirmation of pathophysiological mechanisms of each of these mutations and assessment of their relevance for immune escape extends the scope of this manuscript, the above described mutations might represent an interesting target for future studies of immune escape.

Taken together, the comprehensive molecular and immunological profiling of this unique case of extracranial GBM metastases gives new insights into potential mechanisms of immune escape of GBM. We postulate that the combination of functional impairment of the peripheral immune system, as reflected by a steady increase of exhaustion markers, such as KLRG1 and CD57, and the occurrence of a metastasis with an increased mutational burden, enabled the extracranial dissemination and disease progression while intracranial GBM could be controlled by checkpoint inhibition.

## Materials and methods

### Clinical data

Clinical data and images were analysed after confirmation of peripheral GBM metastasis by two pathologists. The patient signed an informed consent. This study was approved by the local ethics council of the Hamburg chamber for physicians and was performed in accordance with the Helsinki declaration of 1975.

### Histology and immunohistochemistry analysis

Routine histological and immunohistochemical analysis (H&E, GFAP, p53, PD-L1 (clone E1L3N, Cell Signaling), CD3 (clone SP7, Zytomed), CD8 (clone SPI6, DCS)) was performed on paraffin embedded sections using a microtome and automatic staining system (Ventana, Roche Diagnostics).

### PBMC isolation and multicolor flow-cytometry

Peripheral blood was collected into EDTA-containing tubes. Ficoll gradient (PromoCell) centrifugation was performed for the isolation of PBMCs. PBMCs were used after being frozen in RPMI/10% DMSO. Flow-cytometric analysis was performed on PBMCs using multicolor antibody staining as described previously [30]. In addition, immune subpopulations were determined using a TCRγδ-FITC (clone IMMU510, BeckmanCoulter, γδ T cells), CD14-PE (clone RMO52, BeckmanCoulter, monocytes), CD56-ECD (clone N901(NKH-1), BeckmanCoulter, NK cells), CD3-PC-5.5 (clone UCHT1, BeckmanCoulter), CD16-PC-7 (clone 3G8, BeckmanCoulter, monocytes), CD19-APC (clone J3–119, BeckmanCoulter, B cells), CD4-APC-Cy7 (clone RPA-T4, BioLegend), CD45-PacBlue (clone J33, BeckmanCoulter) and for another panel CD25-FITC (clone B1.49.9, BeckmanCoulter), CD3-PC-5.5 (clone UCHT1, BeckmanCoulter), CD8-PC-7 (clone SFCI2ITHY2D3, BeckmanCoulter), CD69-APC (clone FN50, BioLegend) and CD4-APC-Cy7 (clone RPA-T4, BioLegend). Briefly, after F_C_-blocking, samples were stained in flow-cytometry staining buffer (eBioscience) for 45 min at room temperature with the antibody cocktails, washed, and resuspended in buffer prior to analysis. Analysis was performed on a BD LSRFortessa flow-cytometer and using the FACSDiva software (Becton Dickinson).

### CTC / intratumoural immune cell correlation

Formalin fixed paraffin embedded tissue part of an already published cohort in which the peripheral blood was tested for the presence of CTCs, [31] were stained for CD3 (clone SP7, Zytomed), CD8 (clone SPI6, DCS) and CD68 (clone PG-M1, Dako) using a Ventana (Roche Diagnostics). For each tumour, at least ten representative microscopic images were acquired. Images where then automatically quantified using the immuno-ratio plugin for the Fiji analysis software [42].

### Whole exome sequencing and 850 K methylation profiling

Genomic DNA from fresh frozen or paraffin-embedded tumour tissue or peripheral immune cells (> 1 × 10^6^ PBMCs) was isolated using the genomic DNA miniprep kit (innuPREP DNA Mini Kit, Analytic Jena AG). For tumour tissue representative tumour areas were identified from using light microscopy. If necessary, macrodissection was performed. For the metastatic tissue WGS, gDNA was amplified after isolation from paraffin-embedded tissue using the PicoPLEX v2 kit (Takara Bio) as described before [3]. Whole genome sequencing was performed from tumour tissue gDNA using commercial services with the HiSeq X-Ten/PE150 platform using a 350 bp short insert library (BGI Tech Solutions, Hong Kong, China). DNA extraction for methylation analysis was performed from paraffin-embedded tissue using a Maxwell system (Promega). Methylation analysis was performed using the Illumina Infinium MethylationEPIC BeadChip, according to protocols supplied by the manufacturer.

### Data analysis

For whole genome sequencing data, structural variants were called with Manta (v1.6.0) [8]. Strelka (v2.9.10) was used for calling short variants [21]. Both programs were run with default parameters and indel candidates identified by Manta were provided to Strelka as suggested by the authors of the latter program. Variants not passing the tools internal filters were not considered for further analysis. The remaining variants were annotated with Ensembl Variant Predictor (VEP) (v98.2) [28]. Variants annotated with an allelic frequency above 10% in GnomAD were excluded. Other variants were kept if they were predicted to have an impact on protein level or if they had an entry in the COSMIC database (v89). CNA profiles were obtained using Control-FREEC [6] as described before [35]. TMB calculations from WGS data are based on genomic regions which are covered at least 8x in all samples. Methylation data was analysed using the online platform provided by www.molecularneuropathology.org [7]. Statistical analyses of the immunohistochemical stainings was performed with GraphPad Prism. Plots were graphed using GraphPad Prism, R Foundation’s R v2.12, Adobe Illustrator CC 2018.

## Supplementary information

**Additional file 1.** Supplemental Figure 1.

**Additional file 2.** Supplemental Table 1.

## Data Availability

The datasets generated or analysed during the current study are not publicly available due to protection of individual privacy/genetic data protection, but are available after anonymisation from the corresponding author on reasonable request.

## Abbreviations

CD: Cluster of differentiation
CD4: Conventional, non-T_reg_, CD4^+^ T-cells
CNA: Copy number alterations
CNS: Central nervous system
CTCs: Circulating tumour cells
DNA: Deoxyribonucleic acid
GBM: Glioblastoma
HLA: Human leukocyte antigen
IDH1: Isocitrate dehydrogenase 1
KLRG1: Killer cell lectin-like receptor subfamily G member 1
MGMT: O6-Methylguanin-DNA-Methyltransferase
NGS: Next-generation sequencing
PBL: Peripheral blood lymphocytes
PD-1: Programmed death-1
PD-L1: Programmed death ligand-1
T_CM_: central memory T-cell
TCR: T-cell receptor
T_EM_: Effector memory T-cell
T_H_: T-helper cell
TIL: Tumour-infiltrating lymphocytes
TMB: Tumour mutational burden
T_NV_: Naïve T-cell
T_reg_: Regulatory T-cell, CD3^+^ CD4^+^ CD25^high^ CD127^low^
T_TE_: Terminal effector T-cell
T_TM_: Transitional memory T-cell
